# Psychiatry and COVID-19: putting our best foot forward

**DOI:** 10.1192/bjp.2020.90

**Published:** 2020-05-04

**Authors:** Rael D. Strous, Azgad Gold

**Affiliations:** 1 Mental Health Wing, Mayanei Hayeshua Medical Centre, Bnei Brak; and Sackler Faculty of Medicine, Tel Aviv University, Israel; 2 Ambulatory Forensic Psychiatry Unit, Yehuda Abarbanel Mental Health Centre, Bat Yam, Israel

**Keywords:** Ethics, clinical governance, psychosocial interventions, service users, trauma

## Abstract

COVID-19 presents new challenges for psychiatry as clinical management, ethical dilemmas and administrative complications need to be addressed. The psychiatrist should protect the needs and rights of the mentally ill while maximising population health and ensuring solidarity, reciprocity and community well-being for all.

No one yet knows how long COVID-19 will be around. It presents new medical challenges that need to be managed under well-established public health and ethical principles. While all medical branches are confronting the challenges – medical epidemiologists, public health, internal medicine and infectious disease specialists – the field of psychiatry is no less involved. Although psychiatry may appear to be on the periphery of the medical response to COVID-19, critical issues need to be addressed on several fronts.

## Clinical

### Immediate responses

At the local, national and international public health levels, priority is being given to slowing or halting the progress of COVID-19, as well as to combating the medical consequences.^1^ However, other health and patient care needs must be considered, including the ripple effect of the virus and the toll of the upheaval and crisis from a mental health perspective. There are identifiable consequences resulting from the sudden marked change in routine, fear of loss of life, generalised anxiety and economic effects. The circles of effects with collateral damage can often be as important as the immediate effects of infection. There is a need to understand the trade off between, on the one hand, raising awareness of COVID-19 in the media and general consciousness in the community and, on the other, the increased stress and anxiety level that this awareness inevitably causes. The media streams minute-by-minute updates of international statistics and deaths from the novel virus. However, no one knows and no one is measuring morbidity arising from anxiety and enhanced obsessive behaviour. These factors must be taken into consideration at the policy and decision-making level, accompanied by an accumulation of data regarding mental health morbidity due to the viral pandemic. Although this is understandably not always a primary consideration during an international healthcare response to a pandemic, it needs to be a factor. It would also be beneficial to include a mental health professional at the national policy decision-making level to ensure that media coverage reduces anxiety in response to the threat, while maintaining hope and maximising compliance with infection avoidance regulations.

### Resilience and damage control

With the viral crisis having such a harsh impact on the community, mental health specialists may have a unique contribution to make to the development of resilience and optimal coping skills for caregivers and the general community. Advice can be offered on how best to manage isolation and quarantine and how to minimise adverse psychological effects such as frustration, loneliness, anxiety, confusion, anger and family stresses.^2^ It is important to study the effects of isolation in order to best assist those dealing with the phenomenon. Caregivers should be provided with appropriate tools to address the psychological burden that is involved in the onerous COVID-19 treatment environment. Mental healthcare teams with these critical skills need to be recruited by national public health authorities as a critical element in the public health response to COVID-19.

### Short- and long-term mental health consequences

It is reasonable to assume that there will be an aftershock once the threat of COVID-19 is mitigated or over. Psychiatric consequences may include adjustment disorder, worsening obsessive–compulsive disorder, depression, anxiety and even psychosis and increased suicide rates due to the anticipated human and financial loss. Some of these long-term mental health consequences may be minimised by a prudent response by health and other government authorities to the acute viral threat.^3^ It is essential to consider the cost/benefit ratio of the acute response in light of the foreseeable long-term psychiatric effects and concerns. In addition, it seems inevitable that COVID-19 will change perceptions and practices regarding social interactions. Given the appropriateness of social distancing, clinicians should be cautious before pathologising certain behaviours of social avoidance. However, exaggerated social avoidance may require clinical intervention. Lastly, for the foreseeable future at least, COVID-19 will change the traditional setting of interaction with patients, as face-to-face meetings are increasingly replaced by video-based interactions. Relevant clinical, ethical and legal guidelines should be provided for the judicious use of telecommunication technologies.

## Ethics

The COVID-19 crisis raises a number of ethical problems, including several that relate specifically to the psychiatric arena, as follows.

### Involuntary admission and restraint

Can individuals with mental illness be coerced into involuntary psychiatric hospital admission when the only danger that they pose to others is their refusal to respect isolation in acute COVID-19 infection, or their refusal to maintain quarantine when they are only exposed to the coronavirus disease but have not yet contracted the illness? Can patients with minimal or no insight be restrained in a psychiatric ward when the only indication is not respecting isolation to their room? Is restraint appropriate even in a situation where the psychiatric patient is not presenting any clinical symptoms of COVID-19 but has been exposed to a sick patient and only quarantine is required? Meticulous consideration needs to be applied on an individual basis. As a general rule, it may be suggested that the extreme measure of involuntary hospital admission be restricted solely to patients who pose an immediate and serious COVID-19-related threat to themselves or to others. For example, an infected patient with psychosis who is not adhering to isolation guidelines, or a healthy patient who, owing to active psychosis, maintains close uninhibited interaction with a COVID-19-infected individual.

### Managing the risk to mental healthcare providers

People with COVID-19 may die from the illness – latest figures suggest 2–4%.^4^ However, many psychiatric patients have only a mild form of mental illness. Should psychiatric services place the health of mental healthcare providers at risk or in danger in order to manage psychiatric patients with COVID-19? Should psychiatric clinics be closed during the COVID-19 pandemic? As mentioned above, telecommunication technologies may mitigate this ethical dilemma. A reduction in face-to-face interactions with patients seems to be legitimate, especially in the case of infected patients, patients in isolation and high-risk caregivers.

### Redeployment of psychiatrists in general medicine

Psychiatrists are also medical doctors. Given the shortage of medical personnel, should some psychiatrists be diverted from psychiatric treatment and be offered the opportunity to assist in the care of patients in general medicine? This may be appropriate when general medical services become overwhelmed and additional physician services are urgently required.

### The ethics of reporting risk-taking patients

If a psychiatrist is aware that a patient is not respecting the requirements of isolation or quarantine owing to poor insight from psychosis or refusal based on conduct or personality disorder, should the psychiatrist report the individual to the police or other authorities, even though this violates professional trust and patient confidentiality?

### Parity of care and patients’ interests

It is an unfortunate fact that people with psychiatric illness are often the lowest priority in public health decision-making. It is therefore critical that psychiatrists maintain their patients’ right to parity of care even under crisis scenarios of COVID-19. It is also critical that the weakness of psychiatric patients is not exploited regarding any healthcare decisions and that their interests are preserved and upheld.

## Administrative

Various challenges exist for psychiatric administrators during the COVID-19 outbreak. These include the following.

### Protecting patients living in communal facilities

What should be done with patients with chronic psychiatric disorders residing in hostels, assisted living and other non-hospital settings? Should they be left in such surroundings, given the risk for rapid spread of the virus due to poor insight and suboptimal hygienic practices? Ideally, logistic and practical means should be provided to these facilities in order to enable people with chronic psychiatric illness to remain in their familiar environment. If this is not feasible, it is desirable that some of these people be provided with temporary alternative living solutions. If these concerns are left unaddressed, there is a serious concern that these individuals will be unjustifiably diverted to involuntary hospital admission.

### COVID and non-COVID acute wards?

Should specialised psychiatric wards be set up for the management of patients requiring psychiatric hospital admission but who also require isolation or quarantine? Given the estimated high risk of contagion among in-patients on an acute psychiatric ward, the separation between patients' groups, with and without COVID-19 infection, seems appropriate.

## Conclusions

Psychiatry can contribute much to the discourse and community management surrounding the virus onslaught, from unique issues of public health management of chronic mental illness in the community and rehabilitation facilities to reassuring the community in the face of national and international panic and anxiety. With many countries finding themselves in communal crisis, the conflict regarding the individual's rights versus the communal good in ‘protecting the many’ comes into sharper focus. The psychiatrist needs to contribute to this discourse while ensuring maximisation of population health. It is hoped that, along with ensuring public health and its inevitable restrictions, psychiatric input can make a positive contribution, ensuring solidarity, reciprocity and community health for all.

## Supplementary material

For supplementary material accompanying this paper visit http://dx.doi.org/10.1192/bjp.2020.90. click here to view supplementary material
